# Shape and Stability Matter: Enhanced Catalytic Reactions via Sol–gel-Entrapped Catalysts

**DOI:** 10.1007/s41061-022-00415-4

**Published:** 2022-12-21

**Authors:** Rosaria Ciriminna, Mario Pagliaro

**Affiliations:** Istituto Per Lo Studio Dei Materiali Nanostrutturati, CNR, Via U. La Malfa 153, 90146 Palermo, Italy

**Keywords:** Green chemistry, Sol–gel, Heterogeneous catalysis, Fine chemicals, ORMOSIL

## Abstract

The possibility to tune the solid catalyst morphology and the unique chemical and physical stability of organosilica-entrapped sol–gel catalysts allow the application of these catalysts to the synthesis of a wide variety of valued molecules, including polymers, manufactured by the fine-chemical industry. Referring to selected independent research achievements, we provide a practice oriented insight on these materials that will hopefully be useful in new, unified catalysis education aimed to foster the uptake of heterogeneous catalysis in the fine and specialty chemical industry.

**Graphic abstract:**

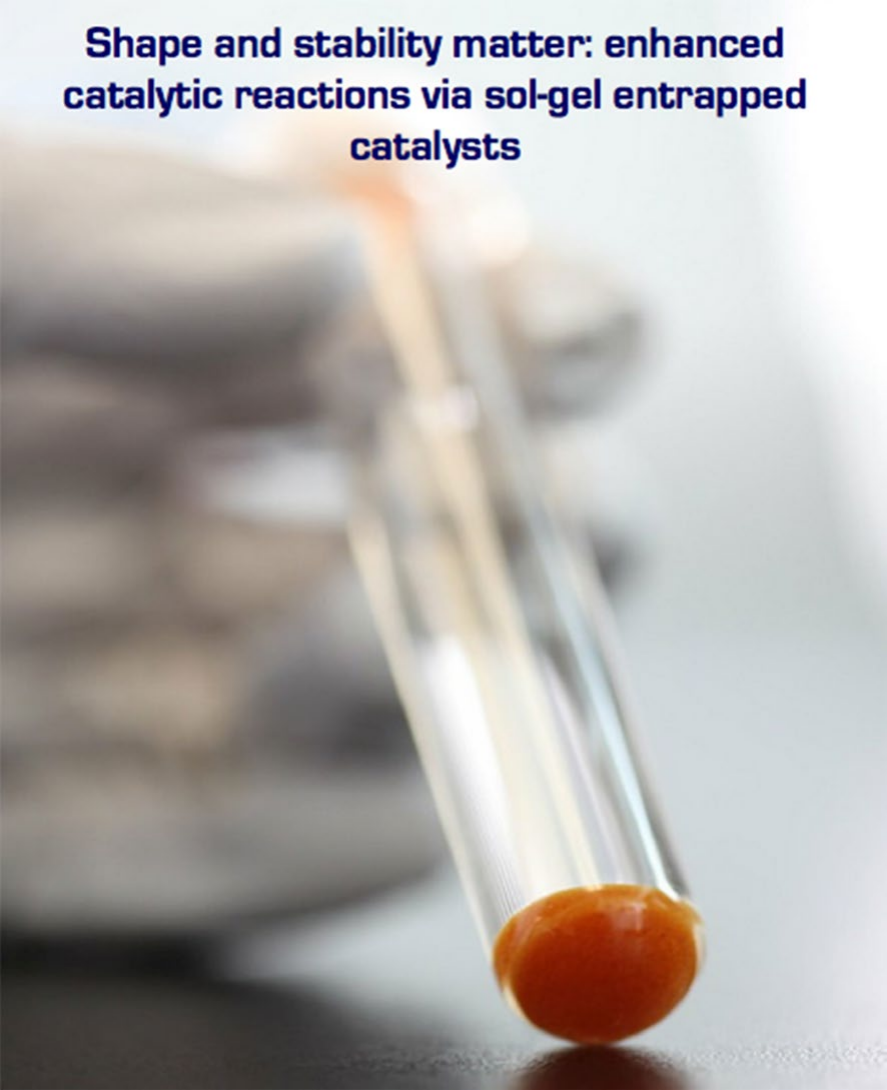

## Introduction

From polymeric arylations [1] through valued pharmaceutical ingredients [2], numerous valued fine chemicals and advanced materials can be synthesized carrying out the catalytic reaction within the inner porosity of sol–gel-entrapped glassy catalysts [3, 4].

According to Hübner, Farina, and de Vries, the fine-chemical industry does not use immobilized transition metal complexes as catalysts due to poor catalyst stability resulting in metal leaching and product contamination [5].

In a series of papers published since the early 2010s, Ananikov’s and Beletskaya’s teams reported the discovery that most catalytic reactions mediated by noble metal nanoparticles, metal salts, and metal complexes in liquid phase actually involve a “cocktail”-type of catalysis in which solid nanoparticles, metal clusters in solution, and soluble metal complexes formed upon metal leaching all take part in catalytic cycles [6, 7].

In the case of a organosilica-entrapped palladium complex sol–gel catalyst, indeed, both homogeneous and heterogeneous catalysis are involved [8]. In cross-coupling reactions, for example, substrates such as iodo-aryls react with the surface of the catalyst to generate hyperactive soluble Pd(II) complexes that, even though present in trace amounts, are responsible for the catalysis observed [9].

Still, the amount of metal leached is so low that it makes leaching not relevant from a practical viewpoint. Furthermore, as shown in the subsequent sections, organosilica-entrapped catalytic materials actually enable new reactions that are not possible with other catalytic materials. The term organosilica above refers to organically modified silicas (ORMOSILs) [10] doped with different chemical species, a truly versatile class of functional materials whose commercial applications today span from biocatalysis [11] through advanced functional coatings [12].

A practical approach to heterogeneous catalysis research (and education) [13], including technical and economic aspects, is critical to enhance the uptake of this green chemistry technology in the fine-chemical industry [14]. In addition, said practice oriented approach to catalysis research and education is useful to foster student motivation and creativity [15]. Hence, referring to recent independent research achievements, in this study we show how the morphology and stability of these glassy catalytic materials provide substantial economic and technical advantages.

As shown in the following section, most sol–gel-entrapped sol–gel catalysts leach ultralow metal amounts, with levels of Pd and Pt in the crude product below the demanding thresholds for active pharmaceutical ingredients. This stability, coupled with the mechanical robustness of glasses sharing huge (several hundred m^2^ g^−1^) surface area and large mesoporosity, makes these materials ideally suited for use in heterogeneously catalyzed syntheses under flow, namely the processes that along with Luque we forecasted to become ubiquitous in the fine chemical industry [16].

Are the mentioned advantages sufficient for the fine chemical industry to switch from homogeneous to heterogeneous catalysis in several key organic processes, from polymeric arylations through cross-coupling and hydrogenation reactions?

## Selected Catalytic Reactions

### Polymeric Arylations

In late 2021, Thompson and coworkers reported that Silia*Cat* DPP-Pd can be successfully employed in the direct arylation polymerization (DArP) for the large-scale and green synthesis of organic semiconducting polymers [1]. The catalyst, an ORMOSIL functionalized with diphenylphosphine ligands bound to Pd(II) in which every Si atom is bound to the C atom of a methyl group [17], was found to be highly efficient and recyclable, affording polymers with molecular weights up to 82,000 g/mol.

**Scheme 1:**
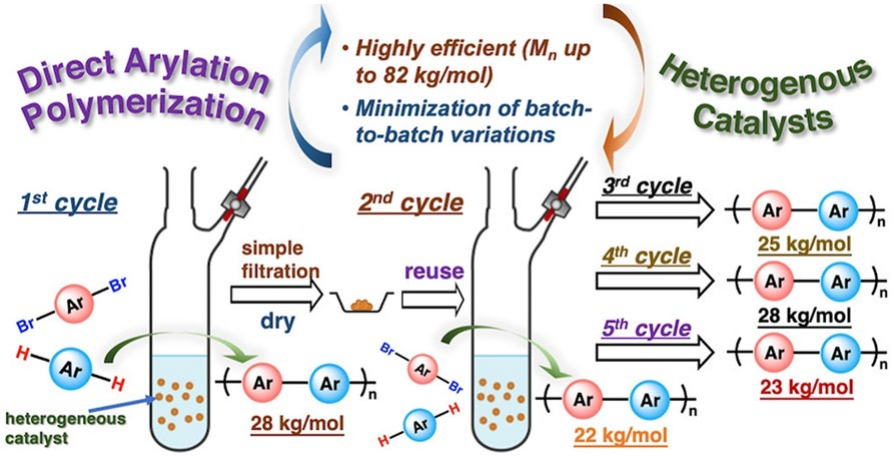
Direct arylation polymerization mediated by Silia*Cat* DPP-Pd. [Reproduced from Ref. 1, with kind permission]

Batch-to-batch variations were further optimized to smoothly obtain polymers without structural disparity, even after five catalyst recycles. It is enough to filter, dry, and reuse the catalyst to obtain consistent polymers with a *M*_*n*_ of 25 ± 2.5 kg/mol (Scheme 1).

**Scheme 2:**
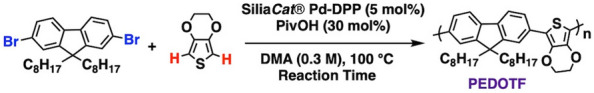
Synthesis of PEDOTF via direct arylation polymerization mediated by Silia*Cat* Pd-DPP [Reproduced from Ref. 1, with kind permission]

In detail, the team used the mild reaction conditions previously reported by Welch’s team for small-molecule double C-H direct arylation of thiophene-based substrate [18], for the synthesis of poly[(3,4-ethylenedioxythiophene-2,5-diyl)-(9,9-dioctylfluorene-2,7-diyl)] (PEDOTF), a polymer with excellent electroluminescent properties that makes it an important candidate for electrochromics and organic light emitting diodes (OLEDs), via direct arylation polymerization (Scheme 2).

Progressively extending the reaction time after the second cycle from 0.5 h first to 2 h and then to 36 h afforded PEDOTF with excellent yields of 91 and 93% and *M*_n_ of consistent molecular weight, with little variation amid batches (average *M*_n_ of 25 kg/mol ± 2.5 kg/mol). This stability, the team concluded, is clearly promising in light of the continuous flow production of conjugated polymers on an industrial scale (Table 1) [1].

**Table 1:** Recycling experiments using Silia*Cat* Pd-DPP under direct arylation polymerization conditions of Scheme 1 [Reproduced from Ref. 1, with kind permission]

	Cycle 1	Cycle 2	Cycle 3	Cycle 4	Cycle 5
Reaction time (h)	0.5	0.5	1.5	2	36
Catalyst recycled (%)		98	94	92	91
*M*_n_ (kg/mol)	28	22	25	28	23
*Đ*	2.1	2.0	2.2	2.1	2.2
Yield (%)	93	92	91	93	92

The team tested two other commercially available heterogeneous Pd catalysts: the polypropylene fiber-supported Pd complex FibreCat Pd, and Pd/C (Tables 2 and 3).

**Table 2:** Recyclability testing of FibreCat Pd and Pd/C under direct arylation polymerization conditions of Scheme 1 [Reproduced from Ref. 1, with kind permission]

		Cycle 1	Cycle 2	Cycle 3	Cycle 4	Cycle 5
Pd FibreCat	Reaction time (h)	0.5	0.5	1.5	3	24
	*M*_n_ (kg/mol), *Đ*	3.5, 1.8	1.8, 1.5	4.2, 1.9	4.4, 1.5	4.7, 1.5
	Yield (%)	73	24	69	37	38
Pd/C	Reaction time (h)	24	24	–	–	–
	*M*_n_ (kg/mol), *Đ*	39.5, 2.43	–	–	–	–
	Yield (%)	92.3	–	–	–	–

**Table 3:** Cost and Pd amount required for the synthesis of 931.9 mg of PEDOTF via DArP mediated by Silia*Cat* DPP-Pd and Pd(OAc)_2_ [Reproduced from Ref. 1, with kind permission]

To synthesize 1.67 mmol of PEDOTF	Amount of Pd (mmol)	Cost ($)
Heterogeneously	0.02	0.65
Homogeneously	0.084	1.79

With FibreCat, catalyst recycling for five batches of PEDOTF was feasible, but with much lower yields and *M*_n_ (1.8–4.7 kg/mol), which was ascribed to the much lower reactivity of FibreCat under the DArP conditions utilized.

Using fresh FibreCat Pd with 24 h of reaction time, PEDOTF of higher *M*_n_ (7.7 kg/mol) was obtained, which should be compared to that obtained (*M*_n_ = 29 kg/mol) using pristine Silia*Cat* DPP-Pd in only 0.5 h.

The activity of Pd/C in the same reaction was due to the leaching of Pd into the solution, which does not allow to recycle the catalyst. In detail, the team found that 24 h was required for the synthesis of PEDOTF via DArP mediated by Pd/C to afford a polymer with *M*_n_ of 39.5 kg/mol and excellent 92.3% yield. However, the catalyst was not reusable after its recovery following the initial run.

The results of the cost analysis (Table 3) showed a substantially lower cost of the heterogeneously catalyzed process. In detail, to synthesize five batches of PEDOTF (a total of 931.9 mg/1.67 mmol) under the optimized batch conditions for the heterogeneous process mediated by Silia*Cat* DPP-Pd employs 66.7 mg of solid catalyst. At the current catalyst price rate ($490/50 g), the amount costs $0.65.

The conventional homogeneous DArP method to synthesize the same total amount of polymer (assuming the same yields and DArP conditions) would require 0.084 mmol (18.7 mg) of Pd(OAc)_2_ where the cost (reagent grade, 98%) totals $1.79.

The amount of Pd required to prepare a total of 1.67 mmol of PEDOTF across five batches, 0.02 mmol, is less than a fourth of that required with the conventional homogeneous approach (0.084 mmol of Pd). In other words, recycling the heterogeneous catalysts in the multi-batch synthesis of the conjugated polymer dramatically reduces the waste of precious Pd metal.

This is particularly relevant for industry because the increasing scarcity and high cost of precious metals such as Pd threatens their supply, making their recovery and recycling via environmentally-friendly process a key objective of today’s chemical research [19].

Besides the negligible loss via leaching, the use of organosilica-entrapped Pd catalysts coupled to the aforementioned new recovery methods will allow easy recovery of palladium from the spent sol–gel catalyst and recycling.

“This work”, Thompson and co-workers concluded, “discloses among the most sustainable conjugated polymer synthesis protocols to date” and “potentially enables access to truly low-cost flow chemistry for industrial-scale conjugate polymer synthesis” [1].

### Continuous Synthesis of Paracetamol

Regardless of being the world’s most prescribed drug, an though being among the most dangerous compounds in medical use [20], analgesic and antipyretic *N*-(4-hydroxyphenyl) acetamide (also called paracetamol or acetaminophen) is no longer produced in western Europe.

In 2008, the last manufacturing facility, located in southern France, was closed as competition from low-production-cost plants in China and India made its production in Europe no longer profitable [21]. The COVID-19 health crisis, however, made it clear how harmful it is for countries to rely on essential drug supplies from foreign countries. Hence, calling for “health sovereignty” [22], France lately became the first western European country where production of acetaminophen will re-start in 2023.

Almost concomitantly, Lecomte-Norrant and Membrat reported a very efficient and nearly waste-free continuous production process of paracetamol [23]. The second key step of the process is the continuous hydrogenation of *p*-nitrophenol over the Silia*Cat* Pd(0) catalyst, an ORMOSIL entrapping Pd 5–6 nm Pd(0) nanoparticles [24], employed at very low load in three reactors of increasing capacity placed in series (with a size ratio of approximately 1:1.5:3).

In this configuration of reactors of increasing size, the fluid flow rate is constant and identical between each reactor, and the heat release due to the highly exothermic hydrogenation reaction is finely controlled leading to very good productivity of the process.

In closer detail, pumping the substrate at 12 ml/min flow rate in three consecutive reactors of 0.1, 0.15, and 0.4 l using, respectively, H_2_ gas pressurized at 20, 12, and 5 bar at slightly increasing temperatures of 100, 110, and 130 °C allows achieving nearly full conversion (98.85%) of *p*-nitrophenol to *p*-aminophenol (Table 4). A remarkable productivity of 3.7 kg/l/day could be obtained.

**Table 4:** Hydrogenation of *p*-nitrophenol to *p*-aminophenol under flow in three consecutive reactors mediated by Silia*Cat* Pd(0): reaction conditions in each reactor [Adapted from Ref. 23, with kind permission]

Parameter	Conditions (R1)	Conditions (R2)	Conditions (R3)
Volume (l)	0.1	0.15	0.4
Flow (ml/min)	12	12	12
Pressure (bar)	20	12	5
Temperature (°C)	100	110	130
Silia*Cat* Pd(0) (%)	0.70%	1.7	2
*p*-nitrophenol entrance (M)	1	0.64	0.16
*p*-nitrophenol exit (M)	0.64	0.16	0.011
Conversion (%)	36	73	93
Global conversion (%)	35.95	83.62	98.85

The new continuous process, in which nitration process is carried out continuously with the substrate dissolved in environmentally friendly solvent ethanol, leads to excellent regioselectivity in favor of the *para* compound (>80% *vs*. 66% of the batch process).

The new process dramatically improves the productivity of the most widely used paracetamol production process based on nitration of phenol with the formation of *p*-nitrophenol followed by hydrogenation and acetylation, suffering from a low yield of phenol nitration reaction, and by the high pressures and long reaction times of the hydrogenation step.

The mesoporous glassy beads of high mechanical strength, large surface area, and high pore volume comprising the sol–gel organosilica-entrapped catalyst are exceptionally well suited for use in flow reactors [25]. On the other hand, Kappe and coworkers highlighted in 2011 how conventional solid catalysts in flow hydrogenations often showed poor stability towards leaching, poor selectivity, and poor activity (low turnover numbers) due to mass transfer kinetic limitations [26].

Finally, the organosilica-entrapped Pd(0) catalyst is not pyrophoric and does not require excluding air from reactions, as it happens with highly pyrophoric conventional Pd/C catalysts. [27]

Self-ignition of spent palladium on a carbon catalyst after hydrogenation reactions is *always* observed with alcoholic solvents [28]. Hence, the use of a non-pyrophoric catalyst such as Silia*Cat* Pd(0) eliminates the risk of spontaneous ignition of the catalyst cake after hydrogenation, providing a key technical advantage when considering the industrial upscale of *p*-nitrophenol hydrogenation *en route* to paracetamol using pressurized H_2_ under flow as reducing agent.

## Practically Relevant Stability

As mentioned in the Introduction, in agreement with Ananikov’s and Beletskaya’s findings on “cocktail-type” catalysis [7], also in the case of the organosilica-entrapped palladium sol–gel catalysts both homogeneous and heterogeneous catalysis are involved [8]. Certain substrates such as iodo-aryls, furthermore, are able to react with the surface of the catalyst to generate hyperactive soluble Pd(II) complexes that are responsible for the catalysis observed, though present in trace amounts [9].

However, from a practical viewpoint, the amount of metal leaching from physically and chemically doped ORMOSIL-entrapped catalysts is so low that it allows to conduct even the synthesis of active pharmaceutical ingredients (APIs) without subsequent product purification.

**Scheme 3:**
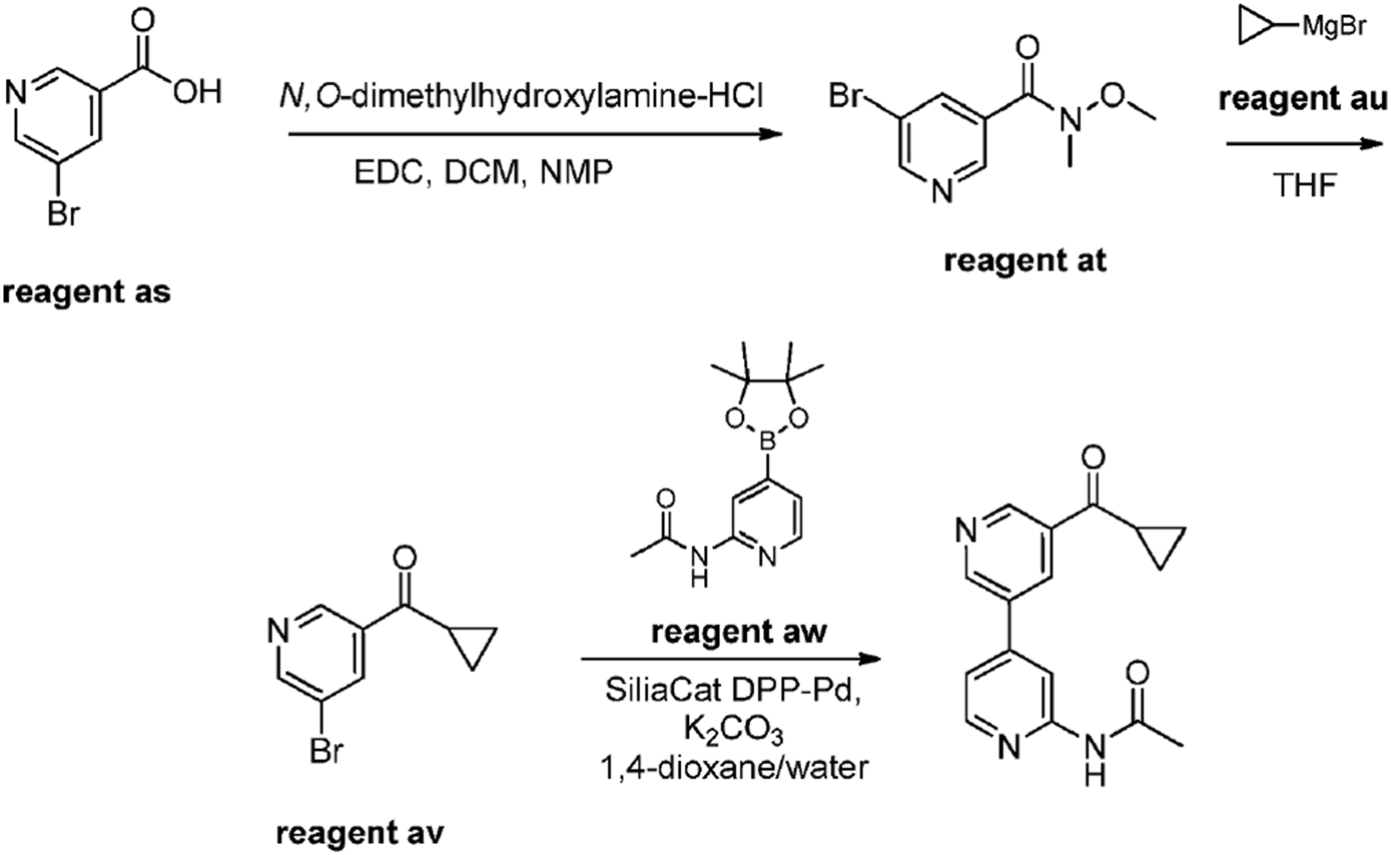
Three-step synthesis of *N*-[5-(cyclopropylcarbonyl)-3,4'-bipyridin-2'-yl]acetamide. The key cross-coupling reaction of is mediated by Silia*Cat* DPP-Pd. [Reproduced from Ref. 29, with kind permission]

For instance, researchers at a pharmaceutical company in Japan used Silia*Cat* DPP-Pd for the synthesis of several complex heteroaryls of pharmaceutical relevance [29]. One example is the reaction of 5-bromopyridin-3-yl)(cyclopropyl)methanone with *N*-[4-(4,4,5,5-tetramethyl-1,3,2-dioxaborolan-2-yl)pyridin-2-yl]acetamide mediated by the solid catalyst in the presence of K_2_CO_3_ as base in dioxane and water heated at 150 °C under microwave irradiation for 40 min. 5-bromopyridin-3-yl)(cyclopropyl)methanone was previously obtained in two steps from 5-bromonicotinic acid converted into 5-bromo-*N*-methoxy-*N*-methylpyridine-3-carboxamide and the latter reacted with cyclopropylmagnesium bromide. Scheme 3 displays the three-step reaction route:

The solvent was removed by rotary evaporation, and the crude compound purified by column chromatography followed by preparative HPLC. No palladium removal was necessary.

The fact that these catalysts enable the synthesis of multiple valued products without requiring isolation and purification from the metal catalyst has been reported by several research groups worldwide.

For example, a joint industry–academia research team in India in 2020 reported a new eco-friendly one-pot sequential synthesis method of quinazolin-8-ol derivatives mediated by Silia*Cat* DPP-Pd without the need of further purification [30].

**Scheme 4:**
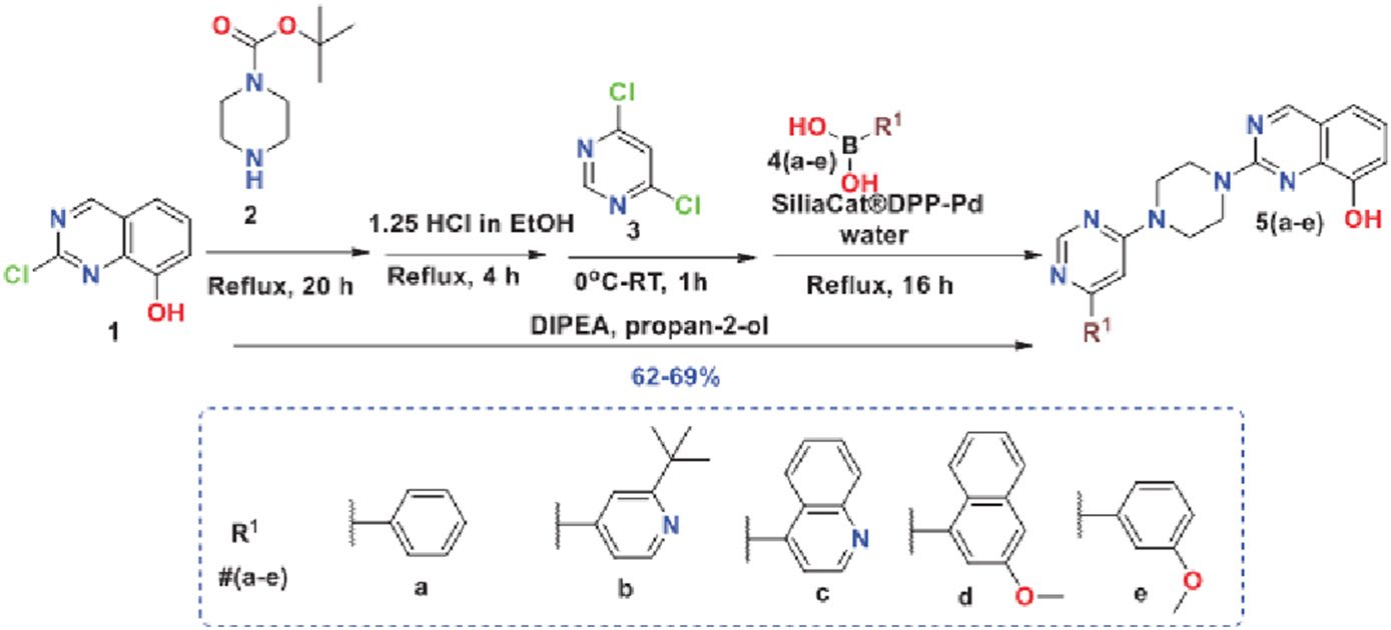
Reaction conditions for one-pot sequential synthesis of quinazoline products **5a–e**. [Reproduced from Ref. 30, with kind permission]

In detail, the method synthesizes quinazoline derivatives via 2-chloroquinazolin-8-ol following an initial substitution of the chlorine atom in commercially available 2-chloroquinazolin-9-ol (**1** in Scheme 4) reacted with Boc-protected piperazine (**2,** Boc is the *N*-*tert*-butyloxycarbonyl amine protecting group). Subsequent de-protection and reaction of B with 4,6-dichloropyrimidine (**3**) gave C which under the Silia*Cat* DPP-Pd Suzuki–Miyaura coupling conditions with boronic acids **4a–e** gave the desired extended quinazoline products **5a–e**.

The reaction is carried out in isopropyl alcohol, a non-toxic solvent used as hand sanitizer, with *N*,*N*-diisopropylethylamine (DIPEA) as mild base. The overall yield obtained in the one-pot synthesis with the final step mediated by the solid catalyst was found to be much higher than in the stepwise, optimized linear synthesis (Table 5).

**Table 5:** Comparison of yield for linear and one-pot synthesis quinazoline derivatives [Reproduced from Ref. 30, with kind permission]

Entry	Product (5a–5e)*	Overall yield (%)	
		Linear synthesis	One-pot synthesis
1	5a	39	69
2	5b	38	65
3	5c	35	67
4	5d	34	62
5	5e	38	68

*See, Scheme 4

Palladium analysis using ICP-MS showed that the palladium content of these compounds, < 10 ppm for the isolated products, was within the low acceptable limit specified International Conference on Harmonisation (ICH) Q3D guidelines on elemental impurities dictating the permissible levels of Pd allowed in the final drug product: 10 ppm as an oral concentration in the drug product, drug substance or excipient [31].

Being route-dependent human toxicants, the platinum group metals, including Pd, Pt, Rh, and Ru, have very low permissible levels allowed in the final drug product.

The newly discovered one-pot synthesis is of significant pharmaceutical relevance. Quinazolines indeed are key APIs used to manufacture numerous drugs, from high-blood-pressure treatments through prevention of neurodegenerative diseases, whose current multistep synthesis suffers from low-to-moderate yields due to the numerous steps involved [32].

Several other APIs can be successfully synthesized over the Silia*Cat* catalysts, ideally under flow conditions. In 2016, another industry–academia research groups in France reported the synthesis of functionalized (7-aza)indoles by flow chemistry in which the three steps – iodination of indole in C-3 position, pyrrole nitrogen protection via *N*-Boc formation, and Sonogashira reaction mediated by Silia*Cat* DPP-Pd contained in a PTFE tubular reactor – are quickly carried out under flow affording high product yields, limited by-product formation, and minimal energy utilization [33].

The team first transferred the iodination and pyrrole nitrogen protection from batch to a continuous process dramatically reducing the reaction time for both the first two steps, proving the efficacy of the 1-min Boc protection reaction also on a 30 mmol amount with 11.1 g h^−1^ throughput.

Having quickly synthesized several 3-iodo-*N*-Boc (aza)indole derivatives, the team showed that the organosilica-entrapped catalyst enables carrying out the Sonogashira reaction in C–3 position using a low amount of heterogeneous catalyst (2.5 mol% of palladium loading) without the need of using a copper co-catalyst (CuI) and a soluble Pd catalyst requiring subsequent product separation from the palladium catalyst.

**Fig. 1:**
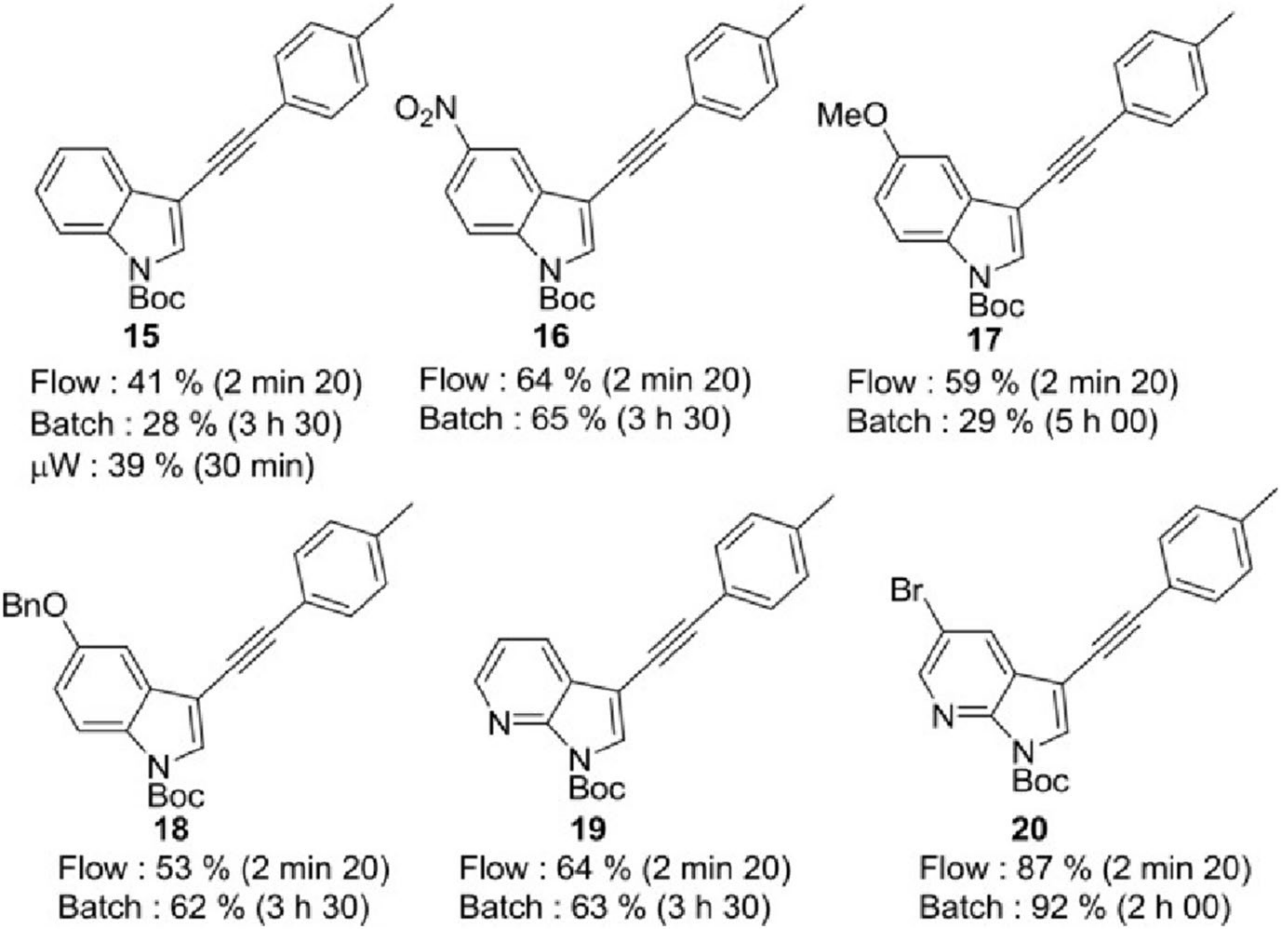
Reaction product and yields in the Sonogashira reaction of several 3-iodo-*N*-Boc (aza)indoles with 4-tolylacetylene as alkyne partner mediated by Silia*Cat* DPP-Pd under batch and flow conditions. [Reproduced from Ref. 33, with kind permission]

The yields of the reaction between numerous 3-iodo-*N*-Boc (aza)indole derivatives with 4-tolylacetylene as alkyne partner displayed in Fig. 1 indicate that under continuous flow conditions, in nearly all cases the reaction afforded higher yields in 140 s only, whereas reaction under batch required from 2 to 5 h.

The method furthermore showed remarkable halogen selectivity. For example, complete discrimination between 5-Br and 3-I atoms on 3-iodo-*N*-Boc (aza)indole bearing a Br atom in position 5 was achieved. Cross-coupling occurred only at the C-3 azaindolic position and derivative **20** (Fig. 1) was isolated in 87% yield with no trace of bromo dehalogenation nor of other azaindolic by-products.

To assess the catalyst stability, the glassy catalytic material contained in the flow reactor was washed on line with DMF (0.02 mL min^−1^, 10 min), and reused in subsequent reaction runs using 100 mg of the same azaindole substrate **14** (5-bromo *N*-Boc-3-iodo(7-aza)indole) with 4-tolylacetylene as coupling reagent.

**Fig. 2:**
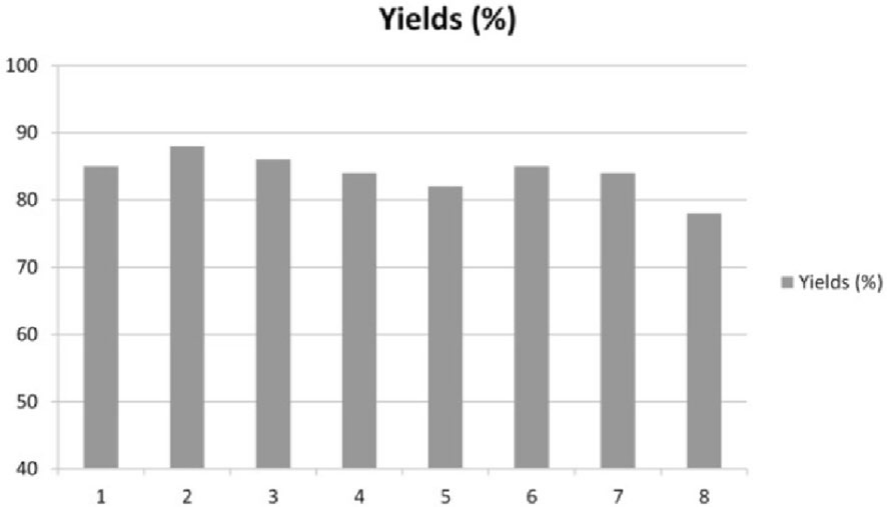
Reusability of Silia*Cat* DPP-Pd in eight consecutive continuous flow reactions of azaindole substrate **14** (100 mg) with 4-tolylacetylene as coupling reagent. [Reproduced from Ref. 33, with kind permission]

Showing evidence of excellent stability on laboratory scale, results in Fig. 2 show that the yield with the same packed-bed reactor of compound **20** synthesized in 700 mg amount was achieved in 140 s in the first seven reaction runs, with a yield decrease observed only at the eighth run.

Azaindoles are important bioactive compounds, with several drugs approved for various diseases being based on the azaindole structural framework. The development of new (and better) synthetic methods for various azaindoles is therefore important in the pharmaceutical industry and in its main supplier, namely the fine-chemical industry [34].

## Outlook and Perspectives

Commenting on the poor uptake of heterogeneous catalysis for cross-coupling reactions found to be an underutilized yet powerful tool for the industry, researchers based at a fine chemical company in the USA recently ascribed such poor uptake to “under-recognition of its benefits such as catalyst recovery, reusability, and low metal leaching” [14].

A decade ago, we argued that the product-oriented fine chemical (and the pharmaceutical) industry will adopt new heterogeneously catalyzed synthetic processes only if economic advantages offered by the new production technology are so large as to cause a quick return on the investment due to the accumulated savings [35]. This is not generally *not* the case for the simple replacement of an homogeneous catalyst with a solid catalyst in the typical batch reactor equipped with reflux condensers comprising the core of the fine chemical industry’s multi-purpose and multi-product plant (MPP) [36].

However, the reduction in production costs achieved when employing heterogeneous catalysts under flow and the reduction in production times is so large to make the switch from MPP to flow reactors employing solid catalysts an inevitable necessity [15]—and this independently of the supported catalyst nature: enzymes [37], single-atom catalysts [38], photocatalysts [39], electrocatalysts [40], or supported metal complexes or metal nanoparticles, as summarized herein for organosilica-entrapped catalysts.

In other words, the slow, but inevitable, uptake of heterogeneous catalysis under flow in the fine-chemical industry originates from technical and economic advantages, and not from the improved environmental “footprint” of the industry’s production processes. Like any other manufacturing organization, fine-chemical companies are selected (and paid) by customers based on product quality, product price, and time of product delivery.

Production of fine chemicals using practically leach-proof heterogeneous catalysts under continuous flow conditions dramatically reduce production cost and production times, while improving product quality (i.e., purity) even under the regulated conditions for the industrial production of APIs [41].

This major shift in chemical production technology, coupled with process automation and continuous monitoring systems, creates the economic and technical conditions to repatriate fine-chemical productions in western Europe, North America, and in former USSR countries, while opening completely new opportunities for API production in Africa and Latin America [42].

Fundamental and applied research in heterogeneous catalysis for synthetic organic chemistry has progressed to the point that by combining a nanomanipulation technique inside a field-emission scanning electron microscope with neural network analysis of selected individual particles it is now possible to identify and select single Pd/C microparticles of exceptional catalytic activity (in cross-coupling reactions) [43].

Similar advances will shortly concern all major catalytic materials. What remains of central relevance to industry is the largely enhanced physical and chemical stability of organic molecules (including organometallic complexes and enzymes) and metal nanoparticles entrapped in silica-based materials [44, 45].

Along with the fact that organosilica does not undergo swelling in any solvent, this leads to the high operational stability required by industry when considering the shift to heterogeneous catalysis under continuous flow conditions (including the case of the regulated production of APIs [41]). Furthermore, the unique possibility to tune the solid catalyst morphology by using the sol–gel template method to produce spherical catalyst microparticles (or submicron particles, if needed) offers another critically important advantage [46]. The catalyst shape, indeed, is often the main performance enabler of a solid catalyst in an industrial reactor, which has recently led catalyst manufacturers to develop new manufacturing processes to access new shapes for commercial catalysts [47].

Again, progress in the field has been rapid and practically relevant. Spherical, rather than irregular, xerogel particles comprised of exceptionally robust silica-alumina functionalized (doped) with noble metal nanoparticles have been developed for the virtually leach-proof hydrosilylation of olefins [48], or the solvent-free full hydrogenation of squalene [49].

Progress will continue to encompass many other ORMOSIL-entrapped metal catalysts based for example on low-cost, abundant metals such as Ni. Recently, for example, Albo and coworkers showed the excellent performance of ORMOSIL xerogels incorporating Ni(II) in mediating the reduction of nitrobenzene to aniline with small amounts of sodium borohydride [50].

Through selected examples of industrially relevant applications of these materials to the synthesis of valued molecules in this study, we have provided a practice oriented insight on the stability and shape aspect of these glassy catalytic materials whose mesoporosity [51] allows to “escape from the microporosity prison” [52], and thus application also to the synthesis of polymers as important as electrochromic conjugated polymers used in organic electronics.

The study, in conclusion, will hopefully also be useful to chemistry educators engaged in new, unified teaching of catalysis [13] aimed to foster practically relevant innovation and uptake of heterogeneous catalysis for fine chemical industrial productions.
